# Using a Health Equity Lens to Evaluate Short-Term Experiences in Global Health (STEGH)

**DOI:** 10.5334/aogh.2926

**Published:** 2020-11-11

**Authors:** Vivian W. L. Tsang, Lawrence Loh

**Affiliations:** 1 Faculty of Medicine, University of British Columbia, Vancouver, BC, CA; 2 Dalla Lana School of Public Health, University of Toronto, Toronto, ON, CA

## Abstract

**Background:**

The growing popularity of short-term experiences in global health (STEGH) has given rise to increasing criticism around their purported benefits and outcomes. With the global health and development community’s growing focus on improving population health and equity worldwide as outlined in the United Nation’s Sustainable Development Goals, there is a growing opportunity to examine and optimize the conduct of STEGH using an outcomes and equity focused lens.

**Objectives:**

This viewpoint aims to develop a framework that can be used to plan and evaluate STEGH on outcomes underpinned by a health equity focus.

**Methods:**

Drawing on logic model theory, the analysis first identifies extant issues and their drivers around the planning, implementation, and evaluation of tradition STEGH (focused on clinical service provision.) The analysis then explores various definitions of health equity, settling on a broad definition around context that promotes health for all as opposed to equity of access to healthcare services. With that definition as the ultimate benchmark of success, the analysis then proposes questions that can be used to determine how and when a STEGH might best be deployed to meet that goal.

**Findings:**

Traditional reliance on process outputs from service-based approaches have historically limited an understanding of if and how STEGH might advance health equity. Using an outcomes-focused approach identifies critical questions around the value of such experiences, when weighed against a broad definition of equity and other key global health themes such as sustainability, cultural humility, and impact. Measuring STEGH against the goal of improving population health status and equity worldwide allows careful consideration of the appropriateness and effectiveness of such efforts on their own and in concert with other interventions.

**Conclusions:**

The extent to which health equity is advanced should be the ultimate metric used to evaluate not only STEGH, but any global health endeavours.

## Background

The popularity of short-term experiences in global health (STEGH) has increased dramatically over the past two decades, raising related questions around their outcomes and effectiveness [1,2]. These efforts typically see visiting volunteers from high-income settings travelling to lower-income settings to provide clinical or surgical care, conduct research, and/or provide community education sessions [3]. The growing popularity of such efforts are driven by continued interest and participation within the medical and public health community, particularly among keen learners and young professionals with an expressed desire to make a difference [3,4].

Mounting criticism has arisen from a growing body of evidence that suggests that the typical conduct of such efforts benefits visiting participants and the organizations that send them more than the communities that welcome them [3,5,6]. This has led to a polarized debate around the practice of STEGH, with some arguing that such efforts should be discouraged and discontinued altogether, while others are staunchly defending such efforts on good intentions and limited benefits observed [5]. Still others advance a pragmatic view that STEGH may play a role in global health and development if appropriately deployed and conducted with an eye towards impact and sustainability [4,6].

Aspirational frameworks, such as the United Nations Sustainable Development Goals, speak to the importance of improving population health status and equity worldwide. Using that as a benchmark, this viewpoint argues that the outcomes of STEGH, as any other global health intervention, should ultimately be evaluated on how well they advance health equity. A focus on outcomes, with equity as the underlying goal, helps determine how STEGH fit in the current global health and development picture, where they might fall short, and what opportunities might exist for them to improve. Notably, this focus is also anticipated to reinforce the concept that access to healthcare is only one determinant in what ultimately improves health equity.

## Towards an Outcomes-Focused Approach in STEGH

An outcomes-focused assessment draws on the logic model framework, which considers program inputs, activities, outputs, and outcomes to plan and evaluate an intervention. First proposed by Wholey in 1979 to clarify the efficiency, effectiveness, and responsiveness of government programs, logic models have since proliferated across numerous sectors, including global health. This conceptual model systematically ties program elements into observable measures at different points in time, with outputs usually being more proximal to the intervention [7,8].

Since many STEGH are traditionally focused on clinical service provision, process outputs are the most commonly used measures of success (e.g. numbers of patients seen or screened, operations conducted, pills distributed, or tests and images completed) [9,10]. Far less common is the measurement of STEGH success against defined health *outcomes* [11]. There are several reasons for this. First, the short-term nature of STEGH limits data collection and the measurement of intermediate outcomes. Programs don’t typically focus on conducting detailed evaluations in a competitive funding environment, preferring to measure success on short-term outputs rather than long-term outcomes [11]. Second, outcomes are often seen at a time period far distal from the time of intervention. This means that proximal outputs are often used as proxy measurements, but in the absence of a clear, evidentiary supported link to a distal outcome, the proximal output alone is not always meaningful in assessing the ultimate long-run impacts of interventions [12]. Finally, the provision of downstream care and service is often preferred, even if it does not address the root cause of ill health. Programs often favour this focus since funders and supporters more easily understand defined outputs as opposed to nuanced, longer term outcomes.

Even absent data that links STEGH activities to health outcomes, focusing on those outcomes can generate hypotheses as to whether traditional service-focused STEGH are driving greater health equity. Typically, the activities undertaken by STEGH depend on which volunteers come (i.e. who wants to go to that locale) and the skills and services they wish to engage [13,14]. This often results in host communities hosting separate, uncoordinated efforts that limits their overall scalability and impact. As an example, clinical STEGH are particularly vulnerable to such impacts on outputs and outcomes if local context and coordination is not considered; one study found that a hospital in the Dominican Republic welcomed STEGH on week to month-long experiences to perform everything from tubal ligation surgery, to dialysis services, to mobile primary clinics, to research and public health education [15].

Efforts that prioritize community development often have more pronounced long-term impacts in addressing the root causes of ill health; clinical interventions, by contrast, usually focus on fixing the disease rather than the conditions that foster its development. Despite this, the focus of most STEGH is largely clinical, since broader community development interventions typically require a longer and more sustained commitment. Clinically focused STEGH may try to address this challenge by incorporating clinical preventive work rather than simply providing downstream care, but this has its own challenges. Preventive work requires careful monitoring, continuity of care, and the deployment of screening or mitigating interventions at the right point in time, which is often at odds with the philosophy of many STEGH that “something is better than nothing at all.”

Finally, a focus on outcomes also requires an examination of the indirect impacts associated with the conduct of STEGH. The application of a logic model often suggests that communities may benefit more significantly from improvements made to local social or economic systems rather than direct healthcare interventions [16,17]. Salient to STEGH, literature suggests such efforts can negatively impact communities indirectly through diverting scarce community resources to host STEGH rather than improving local systems and agencies, or trauma and anguish from receiving culturally incongruent care [2,3,4].

All told, without focusing on prevention or broader community development, most traditional STEGH provide brief, downstream care with significant impacts for communities; this is amplified by rapid cycling between incoming teams. On its face, such a model precludes the delivery of meaningful, lasting, and measurable outcomes.

## To STEGH or Not to STEGH?

The considerations described above highlight the importance of reorienting the planning and evaluation of STEGH towards outcomes. An optimal approach to designing STEGH would ideally start by using a logic model to define the problem, desired outcomes, and a plan to achieve these outcomes based on evidence, data, and context [16,17]. Such an approach, at the very least, would allow sober second thought about whether STEGH are even the right intervention to deploy. A truism in public health reminds that the best trauma system in the world does nothing to address the health impact of motor vehicle collisions; applied to STEGH, the same analogy quickly demonstrates that such efforts are not a panacea. In both limited and well-resourced settings, a singular focus on clinical service often falls short of addressing the antecedent causes of ill health.

There may yet be situations where the deployment of a STEGH may be warranted—but this requires a change from the prevailing paradigm of “something is better than nothing” towards addressing community-identified health priorities. With health equity as the foundation, the first step is to determine key metrics for success and then incorporate STEGH into an overall plan that aims to achieve identified priorities (e.g. STEGH being undertaken together with advocacy efforts by volunteers in their home country that aim to address underlying drivers of global inequities). This develops an understanding that also informs planning, recruitment of the right volunteers with the right skills, and a focus on activities that drive towards outcomes and are undertaken responsibly, impactfully, and in alignment with established community systems.

Most importantly, improved community outcomes should be the primary focus of any STEGH, with learning or development outcomes for participants and sending organizations coming second. Hosting communities should also be empowered to direct work towards priorities that advance health in a lasting manner (e.g. programs that support access to education, clean water, and improved social and economic systems) rather than healthcare provision (e.g. short-term primary care clinics).

A simple series of questions could thus be used to evaluate proposed STEGH:

- What is the desired priority or outcome? (Typically, most STEGH should aim to improve health/promote health equity.)
- Does STEGH have a role in achieving this priority?
  - This can be assessed based on various considerations (e.g. context, data, evidence of impact, etc.).

- If STEGH have a role: what is that role and how does this direct planning, implementation, and evaluation?
- If STEGH does not have a role: do not deploy a STEGH. Instead, determine alternative interventions, if any, that might drive towards the desired outcome (e.g. advocacy for policy change at home; social marketing campaigns to raise awareness and influence decision-makers; direct provision of resources or funding to shore up local capacity; etc.).

Drawing on logic models, this simple sequence of questions helps to determine priority community needs and whether STEGH are placed to address those in a manner that is responsible and impactful [16,17,18]. Considering the current format of STEGH, these questions likely uncover the need for a significant reexamination, particularly as addressing the many determinants that drive poor health and wellbeing must be undertaken at a broad contextual level, rather than through the provision of service. Seen this way, most well-intentioned volunteers might see better results towards their desired outcomes from other interventions instead of participating in STEGH [19].

## The Equity Imperative

In refocusing STEGH planning towards outcomes, this paper has argued that the promotion of greater health equity must be imperative. This is grounded in an understanding that the ultimate goal of any global health intervention, including STEGH, should be to improve health and wellbeing, regardless of any intermediate outputs or outcomes described.

Critically important to this understanding is the definition of health equity, which is sometimes still confused. One common definition traces its lineage from the World Health Organization’s 1948 charter, subsequently built on by the tenets of the Ottawa Charter for Health Promotion, which presents the idea that health is an ideal state and everyday resource for people that must be protected, promoted, optimized, and where necessary, restored [20]. In this school of thought, health equity speaks to the idea that all should reach their full health potential without disadvantage owing to various determinants and circumstances that are governed by context [21].

Stated simply, this definition suggests that advancing health equity requires interventions that create conditions for all to achieve optimal health. This means that access to healthcare is only one part of the puzzle; contexts and environments beyond healthcare must also be shaped to address the underlying factors that perpetuate poor health and inequities.

The second common and competing definition of health equity relates to the idea that there should be equity of access to healthcare services [22]. This has conceptually arisen from key global declarations, starting with Alma-Ata and its call for universal access to essential primary health care services, reaffirmed recently in Astana [23]. While both of these declarations include preventive measures and community development in their definition of primary healthcare, this viewpoint promotes the idea that equity in access to healthcare drives better health. Recent efforts on universal health coverage reflect this thinking, with programs aimed at improving healthcare service access, quality, and financing.

While evidence is clear that primary healthcare provides more cost-effective outcomes than specialist care within healthcare services, literature also clearly demonstrates that access to healthcare services is only one part of what makes people healthy [24]. Certainly, access to healthcare will not sustainably improve the health of communities if the context outside the clinic continues to make them sick. Applying this to STEGH planning and evaluation suggests that such efforts should be measured against the broader concept of health equity, as opposed to improving access to healthcare alone.

## What it Means: For STEGH, and for Global Health and Development

Many people who participate in STEGH go abroad with good intentions—they wish to make a difference for the communities they are welcomed into, to ultimately address the disparities in health status that they are seeing by giving of their time and resources. This means that using the yardstick of greater health equity is even more crucial to ensure that their well-intentioned endeavours are reaching the desired outcomes that they propose, and more importantly, those of the communities that host them.

There are certainly some STEGH that would fare well on this yardstick—cleft palate repair is one that comes to mind. Considering the specific deficit in pediatric surgeons and in particular, pediatric plastic surgeons in many lower-middle-income countries, if properly conducted, with appropriate protocols for follow-up and supports for patients, this life-changing surgery might provide physical and mental health dividends for those patients in the long run [25,26,27].

However, many other traditionally conducted STEGH present limitations that leave them short in driving towards greater health equity. Take, for example, a common example in literature: the STEGH that provides primary care to disadvantaged populations around the world. Stories abound of volunteers visiting communities, setting up ad-hoc clinics in local churches or school, providing medical services like consultations for a variety of ailments, dental services like cleaning of teeth, and even health promotion activities such as education around nutritious foods to eat or brushing teeth [28,29,30,31]. There is almost always Tylenol that can be given for a cold; parasitic medication to reduce the burden of parasites; and laughing and smiling faces as volunteers explain food groups and ways to stay healthy. The process outputs might show that hundreds of patients were seen that day, or that dozens of sessions were delivered.

Yet after the STEGH team leaves, the context remains. Nutrition advice is compromised because the food supply is inconsistent and parasites return, since shelter and hygiene remain inadequate. Escaping poverty remains difficult as entrenched economic and political systems limit job opportunities and community development; perhaps corrupt authorities even target this specific community because they know there are goods to be had—medicines and other charitable items left behind by the visiting team [15].

This example demonstrates the stark reality that the true “health system” is society and the structures that govern it, including healthcare services. It highlights the importance of assessing any intervention with careful identification and pursuit of specific health outcomes. Addressing these challenges requires that STEGH be deployed, not on their own as a panacea, but in a considered manner, as part of a comprehensive strategy to address all the various elements that threaten health equity. It also bears repeating that the comprehensive strategy would need to carefully consider other alternative interventions such as social, political, and economic improvements that could address the broader context that impacts health in the first place as well.

## Conclusion

Healthcare systems in well-resource settings already struggle to focus on health equity beyond access; too often, these systems intervene curatively without resourcing public health and other agencies that work on underlying determinants that might actually be better placed to drive healthy equity.

The same challenges are seen in service-focused STEGH, which are a popular archetype for “making a difference” in global health. Focusing on outcomes, particularly health equity, would improve the deployment of such interventions and better harness the good intentions of participants. Such a focus would also act as a reminder that access to care alone is only one part of achieving health equity, and encourage the deployment of STEGH alongside broader population health efforts to protect, promote, and optimize health in the community, beyond the walls of hospitals and clinics.

Beyond STEGH, the global health and development community and even healthcare practitioners at home would benefit from approaching proposed interventions with one question to start: Does this intervention actually improve health outcomes, and ultimately promote better health equity for all?
